# Mental health impacts in pediatric nurses: a cross-sectional study in tertiary pediatric hospital during the COVID-19 pandemic

**DOI:** 10.1590/1518-8345.5750.3530

**Published:** 2022-05-05

**Authors:** Hingrid Cristiane Silva Robba, Andréa Aoki Costa, Kátia Tomie Kozu, Clóvis Artur Silva, Sylvia Costa Lima Farhat, Juliana Caires de Oliveira Achili Ferreira

**Affiliations:** 1 Universidade de São Paulo, Faculdade de Medicina, Hospital das Clinicas, Instituto da Criança e do Adolescente, Divisão de Enfermagem, São Paulo, SP, Brasil.; 2 Universidade de São Paulo, Faculdade de Medicina, Departamento de Pediatria, São Paulo, SP, Brasil.

**Keywords:** COVID-19, Mental Health, Anxiety, Depression, Nurses, Pediatric, Burnout, Professional, COVID-19, Saúde Mental, Ansiedade, Depressão, Enfermeiras Pediátricas, Esgotamento Profissional, COVID-19, Salud Mental, Ansiedad, Depresión, Enfermeras Pediátricas, Agotamiento Profesional

## Abstract

**Objective:**

to assess mental health issues in pediatric nurses during coronavirus pandemic in 2019.

**Method:**

cross-sectional study was conducted with pediatric nurses at the *Instituto da Criança e do Adolescente* based on online self-rated survey about clinical practice and mental health impact during COVID-19 pandemic. Validated self-reported scales for anxiety, depression and burnout were used for assessing these professionals.

**Results:**

107/298 (36%) nurses answered, 90% were female, median age was 41(23-64) years, 68% worked with adolescents, 66% in frontline. Burnout, anxiety and moderate/severe depression occurred in 65%, 72% and 74%, respectively. Lack of standardized treatment protocol for nurses (27%vs.10%, p=0.049), moderate/severe depression (74% *vs*. 16%, p=0.002) and burnout (82% *vs*. 58%, p=0.01) were significantly higher in pediatric nurses with anxiety compared to those without. Pediatric nurses that worked with adolescents compared to those that did not showed higher frequency of burnout in the former group (77% *vs*. 32%, p=0.0001). Multivariable analysis revealed that adequate quarantine adherence increased the presence of anxiety in 4.6 times [OR4.6(CI 1.1-20.2), p=0.04].

**Conclusion:**

most pediatric nurses who had worked in the frontline of COVID-19 were under precarious conditions, working with reduced team, and with an expressive changes in their monthly income. Current anxiety was a relevant issue, burnout was also an important mental condition for these professionals, reinforcing culture of good teamwork, collaboration practices and psychological/psychiatric approach.

## Resumo

**Objetivo:**

avaliar problemas de saúde mental em enfermeiros pediátricos durante a pandemia causada pelo coronavírus 2019.

**Método:**

estudo transversal realizado com enfermeiros pediátricos do Instituto da Criança e do Adolescente, por meio de uma pesquisa online de autoavaliação sobre prática clínica e impacto na saúde mental, durante a pandemia de COVID-19. Foram avaliadas escalas de autoavaliação validadas para ansiedade, depressão e *burnout*.

**Resultados:**

107/298(36%) enfermeiros responderam, dos quais 90% eram do sexo feminino, a mediana de idade atual era 41(23-64) anos, 68% trabalhavam com adolescentes, 66% trabalhavam na linha de frente. *Burnout*, ansiedade e depressão moderada/grave ocorreram em 65%, 72% e 74% dos enfermeiros, respectivamente. Falta de protocolo de tratamento padronizado nas enfermarias (27% *vs*. 10%, p=0,049), depressão moderada/grave (74% *vs*. 16%, p=0,002) e *burnout* (82% *vs*. 58%, p=0,01) foram significativamente maiores em enfermeiros pediátricos com ansiedade, em comparação com enfermeiros sem essa condição. Os enfermeiros pediátricos que trabalhavam com adolescentes apresentaram maior frequência de *burnout*, quando comparados aos que não trabalhavam com esse grupo (77% *vs*. 32%, p=0,0001). A análise multivariada revelou que o cumprimento adequado da quarentena aumentou a presença de ansiedade em 4,6 vezes [OR 4.6(IC 1,1-20,2), p=0,04].

**Conclusão:**

a maioria dos enfermeiros pediátricos atuava na linha de frente da COVID-19, em condições precárias, trabalhando com equipe reduzida e enfrentando perdas expressivas de renda. A ansiedade atual foi um tema relevante e o *burnout* também foi uma condição mental importante para esses profissionais, reforçando a cultura do bom trabalho em equipe, das práticas de colaboração e do cuidado psicológico/psiquiátrico.

## Resumen

**Objetivo:**

evaluar los problemas de salud mental en enfermeros pediátricos durante la pandemia del coronavirus 2019.

**Método:**

estudio transversal realizado con enfermeros pediátricos del *Instituto da Criança e do Adolescente* mediante una encuesta de autoevaluación en línea sobre la práctica clínica y el impacto en la salud mental durante la pandemia de COVID-19. Se evaluaron escalas de autoevaluación validadas para ansiedad, depresión y *burnout*.

**Resultados:**

respondieron 107/298 (36%) de los enfermeros, de los cuales 90% eran del sexo femenino, la mediana de edad actual fue de 41 (23-64) años, 68% trabajaban con adolescentes, 66% trabajaban en primera línea. *Burnout*, ansiedad y depresión moderada/grave ocurrieron en 65%, 72% y 74% de los enfermeros, respectivamente. Falta de protocolo de tratamiento estandarizado en las enfermerías (27% vs. 10%, p=0,049), depresión moderada/grave (74% vs. 16%, p=0,002) y *burnout* (82% vs. 58%, p=0,01) fueron significativamente mayores en enfermeros pediátricos con ansiedad en comparación con enfermeros sin esta condición. Los enfermeros pediátricos que trabajaban con adolescentes presentaron mayor frecuencia de *burnout* en comparación con los que no trabajaban con adolescentes (77% *vs*. 32%, p=0,0001). El análisis multivariado reveló que el adecuado cumplimiento de la cuarentena aumentó 4,6 veces la presencia de ansiedad [OR 4.6(IC 1,1-20,2), p=0,04].

**Conclusión:**

La mayoría de los enfermeros pediátricos trabajaban en la primera línea de la COVID-19, en condiciones precarias, trabajando con un equipo reducido y enfrentando importantes pérdidas de ingresos. La ansiedad actual fue un tema relevante y el *burnout* también fue una condición mental importante para estos profesionales, reforzando la cultura del buen trabajo en equipo, las prácticas colaborativas y la atención psicológica/psiquiátrica.

**Highlights**

(1) Pandemic infectious diseases may promote psychological changes in health care workers.

(2) Anxiety was a relevant issue reported by pediatric nurses during COVID-19 pandemic.

(3) Burnout was an important mental condition for professionals who had been working with adolescents.

(4) Hospitals must incorporate an appropriate health program for professionals.

## Introduction

Coronavirus pandemic (COVID-19) in 2019, caused by severe acute respiratory syndrome coronavirus 2 (SARS-CoV-2) infection, have changed healthcare workers lives. 

Pandemic infectious diseases may promote psychological changes in health care workers, especially women and nurses. Indeed, preexisting health conditions, feelings of vulnerability, social distancing, concerns about family members may increase anxiety, depression and burnout for these health professionals^1^ ^-^ ^2^.

Children and adolescents have infrequent COVID-19, however, they may present moderate to severe conditions requiring hospitalization^3^ ^-^ ^5^. Pediatric nurses have been working tirelessly to care for COVID-19 patients, many of them need to stay away from their homes for prolonged periods, as they are afraid they may be putting their own families at risk of infection, impacting their mental and physical health^6^. In addition, number of deaths, changes in monthly income, sleep deprivation and sedentarism during this pandemic may influence self-reported anxiety and depression for nurses^7^ and general practitioners, particularly for nurses who treats and care for adult patients around the word^7^ ^-^ ^13^.

However, to our knowledge, these issues were not systematically assessed in pediatric nurses during the COVID-19 pandemic, particularly in professionals of a tertiary hospital that follows-up children and adolescents with chronic and severe conditions. 

Therefore, the aim of the present study was to assess demographic data, work-related conditions, and mental issues of pediatric nurses during pandemic COVID-19. We also compared demographic and issues related to COVID-19 data in pediatric nurses with and without anxiety, with and without depression, as well as pediatric nurses that worked with adolescents compared to those that did not work with teenagers, as there is a high percentage of patients in this age group being treated at the mentioned institution.

## Method

From July to October 2020, a cross-sectional study was carried-out with pediatric nurses who were regularly working during the period studied, anchored by an online questionnaire about physical and mental health impacts during COVID-19 pandemic. All pediatric nurses who had been working at the *Instituto da Criança e do Adolescente do Hospital das Clinicas da Faculdade de Medicina* from the *Universidade de São Paulo*, a large university hospital in São Paulo metropolitan area, Brazil (n=298) were invited to participate. A total of 107 (36%) pediatric nurses answered our online survey, and therefore, they were selected as participants.

This anonymous survey was carried out using Research Electronic Data Capture (REDCap) tool to support data capture for research studies. Ethics Committee of our university hospital have approved our research (under the number 4.037.335), and the Informed Consent Form was signed by all participants in the beginning of survey. The online survey was sent to these professionals and at least six emails were sent to all of them to improve the response rate.

The survey included questions about clinical practice, as well as physical and physiological health problems during COVID-19 pandemic. Estimated time for responses of all survey was approximately 15 minutes. These questions were categorized in multiple choice-response, dichotomous (yes and no) or based on visual analogue scale (VAS) (ranged from 0-10) scores, and included:

- Demographic data of pediatric nurses (current age and gender).
- Pediatric nurse in remote attendance (telemedicine, cell phone orientations or both).
- Whether they were working with adolescents (yes/no).
- Whether they were working in the frontline of COVID-19 (yes/no).
- Number of pediatric patients during COVID-19 (decreased, increased or remained the same).
- Number of pediatric patients with suspected COVID-19.
- Suspected or confirmed death in COVID-19 patients.
- Changes in monthly income of pediatric nurses during COVID-19 (decreased, increased or remained the same).
- Molecular and serological methods performed by nurses [real time - reverse transcription polymerase chain reaction (RT-PCR), serology for SARS-CoV-2) infection or both] (yes/no).
- Seasonal influenza vaccination prescribed for pediatric patients during the pandemic (yes/no).
- Seasonal influenza vaccination received by nurses during the pandemic (yes/no).
- General supportive care availability for COVID-19: pediatric nurses, other pediatric health professionals, appropriate personal protective equipment, standardized treatment protocols for children and adolescents with COVID-19, pediatric COVID-19 ward and pediatric intensive care units availability.
- Pediatric nurses reported feelings: feelings of apprehension, panic, tranquility, optimism, satisfaction and dissatisfaction.
- The most important impact of pandemic in pediatric nurse: none, change in their monthly wages, psychological health issues, family concerns or preexisting health comorbidity in pediatric nurses (yes/no).
- Preexisting health conditions of pediatric nurses: asthma, diabetes mellitus, cardiovascular condition, respiratory condition, arterial hypertension, renal insufficiency, obesity, tobacco use or others.
- Changes in body weight reported by pediatric nurses during pandemic: increased, decreased, no change.
- Adequate quarantine adherence (yes/no).
- Physical activity according to VAS score, ranging from 0 (without any physical activity) to 10 (strong physical activity daily).
- Night time sleep abnormalities according to VAS score, ranging from 0 (no abnormality) to 10 (severe insomnia).
- Fear of COVID-19 according to VAS score, ranging from 0 (no fear) to 10 (with extreme fear).
- Frequency of alcohol intake in the past 15 days (never, rarely: once a week, sometimes: around once a week, regularly: up to twice a week, frequently: 3 to 6 times a week or every day).
- Total amount of alcohol intake [one shot (350ml of beer), or a glass of wine or 40 ml of vodka/whisky]: 1 to 2 shots, 3 to 4 shots, 5 to 6 shots or more than 6 shots.
- Cigarettes number (no tobacco use, 1 to 5 cigarettes/day, 5-10 cigarettes/day, 10-20 cigarettes/day or more than 20 cigarettes/day).
- Smoking history and return to smoking during the past 15 days (yes/no).
- Frequency of smoking marijuana/*cannabis* in the past 15 days: never, rarely: once a week, sometimes: around once a week, regularly: up to twice a week, frequently: 3 to 6 times a week or every day.
- Frequency of opioids use (morphine, codeine, tramadol) in past 15 days: never, rarely: once a week, sometimes: around once a week, regularly: up to twice a week, frequently: 3 to 6 times a week or every day.
- Frequency of sleeping pills usage in the past 15 days: never, rarely: once a week, sometimes: around once a week, regularly: up to twice a week, frequently: 3 to 6 times a week or every day.

Burnout symptoms were measured by a single-item burnout measurement based on the Maslach Burnout Inventory Emotional Exhaustion (MBI:EE)^14^. Participants answered the following question: “In general, based on your definition of exhaustion, how would you rate your level of exhaustion?” the chosen answer scored on an ordinal scale of five categories: 1=*“I enjoy my work. I have no symptoms of burnout”*; 2 =*“Occasionally I am under stress, and I don’t always have as much energy as I once did, but I don’t feel burned out”*; 3 =*“I am definitely burning out and have one or more symptoms of burnout, such as physical and emotional exhaustion”*; 4 = “*The symptoms of burnout that I’m experiencing won’t go away. I think about frustration at work a lot”*; and 5 =*“I feel completely burned out and often wonder if I can go on. I am at the point where I may need some changes or may need to seek some sort of help”*. Score ≤2 was considered as no symptoms of burnout and ≥3 was considered as presence of 1 or more burnout symptoms^14^ ^-^ ^15^.

Anxiety and depression were considered as dependent variables. The validated self-reported scores for depression and anxiety in Portuguese language were also evaluated for pediatric nurses based on responses based on their previous 15 days. Patient Health Questionnaire (PHQ-9) scale comprised nine questions using four-point Likert-scale ranging from 0 (not at all) to 3 (almost every day) for each question, and score ranged from 0 to 27. Total score of pediatric nurses was characterized as absent (scores 0-4), mild (scores 5-9), moderate (scores 10-14), moderate to severe depression (scores 15-19) and severe depression (scores 20-27)^16^. Generalized Anxiety Disorder (GAD-7) scale comprised seven questions using four-point Likert-scale, ranged from 0 (not at all) to 3 (almost every day) for each question. The total score of pediatric nurses was considered and divided in two groups: without anxiety (scores ≤ 4) and with anxiety (scores ≥ 5)^17^.

### Statistical analysis

Data were presented as median (minimum to maximum values) or mean ± standard deviation for continuous variables according to the Shapiro-Wilk test. Data were showed in number (percentage) for categorical variables. Mann-Whitney test or Student’s t-test were used to compare continuous variables, and Fisher exact test or Pearson chi square test were used for categorical variables, as indicated and *p* value <0.05 was considered as significant. Logistic regression models were used to identify possible factors that would increase the chance of anxiety. Variables with a level of significance less than or equal to 20% in the univariable model were included in the multivariable model as independent variables. The results of the final model are presented as odds ratios (ORs) and 95% confidence intervals (CIs). For all statistical tests, the significance level was set at 5% (p < 0.05). SPSS software, version 22 (IBM Corporation, Armonk, NY, USA) was used for analysis.

## Results

The overall response rate was 107/298 (36%) and 90% were female. Table 1 shows demographic and COVID-19 data reported by pediatric nurses during pandemic. Working in the frontline of COVID-19 was reported by 66% of them, the median age was 41 years (23-64), 68% worked with adolescents and 66% worked in the COVID-19 frontline. Deaths in patients with confirmed or suspected COVID-19 were reported by 47%. Changes in monthly wages during the pandemic COVID-19 and feelings of apprehension were informed by 84% and 82% of pediatric nurses, respectively. Anxiety, burnout and moderate/severe depressive symptoms were reported by 65%, 74% and 72%, respectively and emotional impact of pandemic was related by 58% of pediatric nurses (Table 1).

**Table:** 

Variables reported by pediatric nurses	Pediatric nurses (n=107)
**Demographic data**	
Female gender	96 (90)
Current age, years	41 (23-64)
Local of practice	60 (56)
Emergency and intensive care units	23 (21)
Inpatients Unit	24 (22)
Ambulatory and administrative	13 (12)
Working in the frontline of COVID-19	71 (66)
Working in two or more hospitals	6 (6)
**Remote care provided n=98**	
None	91 (85)
Cell phone orientation	4 (4)
Telemedicine	3 (3)
Decreased number of patients during pandemic	54 (50)
Deaths in patients with confirmed or suspected COVID-19	51 (47)
Changes in monthly income during pandemic COVID-19 by pediatric nurses	90 (84)
Seasonal influenza vaccination	104 (97)
**Molecular and serological methods performed by nurses**	
Real-time RT-CPR* positive	4 (4)
Real-time RT-CPR* negative	44 (41)
Quick test negative	24 (22)
No test	35 (33)
Confirmed COVID-19 in pediatric nurses	4 (4)
**General supportive care availability for COVID-19**	
Lack of pediatric nurses	49 (46)
Lack of other pediatric healthcare workers	62 (58)
Lack of appropriate personal protective equipment	22 (20)
Lack of standardized pediatric treatment protocol	23 (21)
Lack of pediatric Intensive care unit	16 (15)
**Reported feelings**	
Feelings of apprehension	88 (82)
Feelings of dissatisfaction	24 (23)
Feelings of optimism	20 (19)
Feelings of panic	11 (10)
**Most important impact of pandemic**	
Changes in monthly income	7 (6)
Emotional	62 (58)
Family concerns	34 (32)
**Preexisting health condition**	
None	61 (57)
Arterial hypertension	22 (21)
Obesity	17 (16)
Diabetes	7 (6)
Smoking	5 (5)
Adequate quarantine adherence	67 (63)
Fear of COVID-19 (VAS^†^ scale 0-10)	7 (0-10)
Physical activity (VAS^†^ scale 0-10)	0 (0-10)
Sleep deprivation (VAS^†^ scale 0-10)	5 (0-10)
Anxiety	69 (65)
Mild	42 (39)
Moderate to severe	27 (25)
Depression	77 (72)
PHQ-9^‡^ score 5-9 - mild	25 (23)
PHQ-9^‡^ score 10-14 - moderate	29 (27)
PHQ-9^‡^ score 15-19 - moderate to severe	14 (13)
PHQ-9^‡^ score >20 - severe	9 (8)
Burnout	79 (74)
Alcohol consumption	53 (49)
Tobacco use	9 (8)
Return to smoking during COVID-19 pandemic	7 (6)
*Cannabis* use	1 (0,9)
Sleeping pills consumption	17 (16)
Working with adolescents as patients	73 (68)
Nursing practice with adolescents, years	10 (0.8-36)

Results are presented in n (%) and median (minimum to maximum values); *RT-PCR = Reverse Transcription Polymerase Chain Reaction; ^†^VAS = Visual Analogue Scale; ^‡^PHQ-9 = Patient Health Questionnaire

Table 2, below, presents demographic and COVID-19 data reported by pediatric nurses during pandemic according to GAD-7 scale for anxiety. Moderate/severe depression (PHQ-9 score ≥10) (74% *vs*. 16%, p=0.002) and burnout (82% *vs*. 58%), p=0.01 were significantly higher in pediatric nurses with anxiety compared to those without this condition, while mild depression (PHQ-9 score ≥5 and ≤9) (26% *vs*. 60%, p=0.016) and no depression (PHQ-9 score was≤ 4) (10% *vs*. 61%, p< 0.001) were more prevalent in nurses without anxiety.

**Table 2:** Demographic and coronavirus infectious disease in 2019 (COVID-19) data reported by 107 Pediatric Nurses during pandemic according to professionals with anxiety (GAD-7^*^ score ≥ 5) and without anxiety (GAD-7^*^ score ≤ 4)**.** São Paulo, SP, Brazil, 2020

Variables of pediatric nurses	With anxiety (n=69)	Without anxiety (n=38)	*P*
**Demographic data**			
Current age, years	41 (23-64)	41 (28-60)	0.147
Female gender	64 (93)	32 (84)	0.192
Working in the frontline with suspected COVID-19	49 (71)	22 (20)	0.202
Changes in monthly income			0.789^†^
None	56 (81)	34 (89)	0.408
Decrease of up to 20%	7 (10)	3 (8)	1.0
Decrease between 21 to 50%	3 (4)	1 (3)	1.0
Decrease of more than 50%	3 (4)	0 (0)	-
**Molecular and serological methods performed by nurses**			
None	20 (29)	15 (39)	0.288
Real time RT-CPR^‡^	33 (48)	15 (39)	0.425
Serology for SARS-CoV2^§^ infection	16 (23)	8 (22)	1.000
Confirmed COVID-19 in pediatric nurses	3 (4)	1 (2)	1.000
**General supportive care availability for COVID-19**			
Lack of pediatric nurses	33 (48)	16 (42)	0.685
Lack of other pediatric healthcare workers	43 (62)	19 (50)	0.227
Lack of appropriate personal protective equipment	17 (25)	5 (13)	0.213
Lack of standardized treatment protocol in pediatric ward	19 (27)	4 (10)	0.049
Lack of pediatric Intensive care unit	12 (17)	4 (10)	0.407
**Reported Feelings**			
Feelings of apprehension	60 (87)	28 (74)	0.113
Feelings of panic	8 (11)	3 (8)	0.743
Feelings of dissatisfaction	19 (27)	5 (13)	0.097
Feelings of optimism	14 (20)	6 (16)	0.615
Adequate quarantine compliance	47 (68)	20 (52)	0.144
Fear of COVID-19 (VAS^||^ scale 0-10)	7 (0-10)	7 (1-10)	0.975
Physical activity (VAS^||^ scale 0-10)	0 (1-10)	0 (1-10)	0.955
Sleep deprivation (VAS^||^scale 0-10)	5 (0-10)	5 (0-10)	0.928
**Pre-existing health condition**			
Obesity	11 (16)	6 (16)	1.000
Arterial hypertension	15 (22)	4 (10)	0.190
Diabetes mellitus	4 (6)	3 (8)	0.697
Smoking	3 (4)	2 (5)	1.000
Impact of pandemic			0.389^†^
Decrease of income	5 (7)	2 (5)	1.0
Mental health	43 (62)	19 (50)	0.228
Family concerns	19 (27)	15 (39)	0.278
PHQ-9^¶^ score	9 (0-24)	9 (0-17)	0.183
PHQ-9^¶^ score ≥10	46/62 (74)	6/15 (16)	0.002
PHQ-9^¶^ score 5-9	16/62 (26)	9/15 (60)	0.016
PHQ-9^¶^ score ≤4	7(10)	23 (61)	<0.001
Burnout	57 (82)	22 (58)	0.01
Tobacco use	7 (10)	2 (5)	0.486
Return to smoking	5 (7)	2 (5)	1.000
Cannabis use	1 (1)	0 (0)	1.000
Sleeping pills consumption	16 (23)	3 (8)	0.064
Working with adolescents as patients	46 (67)	27 (71)	0.671

Results are presented in n (%) and median (minimum to maximum values); *GAD-7 = Generalized Anxiety Disorder; ^†^Pearson Chi Square; ^‡^RT-PCR = Reverse Transcription Polymerase Chain Reaction; ^§^SARS-CoV2 = Severe Acute Respiratory Syndrome Coronavirus 2; ^||^VAS = Visual Analogue Scale; ^¶^PHQ-9 = Patient Health Questionnaire

Table 3 shows demographic and COVID-19 data reported by pediatric nurses during pandemic according to PHQ-9 scale for depression. Lack of pediatric nurses (60% *vs*. 33%, p=0.006), burnout (86% *vs*. 62%, p=0.004), GAD-7 score ≥ 5 (88% *vs*. 42%, p=0.0001) and consumption of sleeping pills (33% *vs*. 4%, p=0.0001) were significantly higher in pediatric nurses with depression compared to those without this condition. Regarding nurses who worked with adolescents, the prevalence of PHQ-9 ≥ 10 (49% *vs*. 47%, p=0.83), GAD-7 ≥ 5 (63% *vs*. 68%, p=0.67) was similar to nurses who did not work with adolescent. Burnout was significant more prevalent in former group (77% *vs*. 32% (p<0.001).

**Table 3:** Demographic and coronavirus infectious disease in 2019 (COVID-19) data reported by Pediatric Nurses during pandemic according to professionals with moderate/severe depression (PHQ-9^*^ score ≥ 10) and without/mild depression (PHQ-9^*^ score < 10). São Paulo, SP, Brazil, 2020

Variables of pediatric nurses	Moderate/severe depression (n=52)	Without/mild depression (n=55)	*P*
**Demographic data**			
Current age, years	41 (23-64)	41 (24-63)	0.703
Female gender	49 (94)	47 (85)	0.203
Working in the frontline with suspected COVID-19	35 (67)	36 (65)	1.000
Changes in monthly income			0.287^†^
None	42 (81)	48 (87)	-
Decrease of up to 20%	5 (10)	5 (9)	-
Decrease between 21 to 50%	2 (4)	2 (4)	-
Decrease of more than 50%	3 (6)	0 (0)	-
**Molecular and serological methods performed by nurses**			
None	16 (31)	19 (34)	0.686
Real time RT-CPR^‡^	24 (46)	24 (44)	0.847
Serology SARS-CoV2^§^ infection	12 (23)	12 (22)	1.000
Confirmed COVID-19 in pediatric nurses	2 (4)	2 (4)	1.000
**General supportive care availability for COVID-19**			
Lack of pediatric nurses	31 (60)	18 (33)	0.006
Lack of other pediatric healthcare workers	35 (67)	27 (49)	0.077
Lack of appropriate personal protective equipment	14 (27)	8 (14)	0.151
Lack of standardized treatment protocol ward	14(27)	9 (16)	0.240
Lack of pediatric Intensive care unit	7 (13)	5 (9)	0.549
**Reported Feelings**			
Feelings of apprehension	45 (86)	43 (78)	0.316
Feelings of panic	8 (15)	3 (4)	0.116
Feelings of dissatisfaction	13 (25)	11 (20)	0.644
Feelings of optimism	8 (15)	12 (22)	0.461
Adequate quarantine compliance	31 (60)	35 (64)	0.695
Fear of COVID-19 (VAS^||^ scale 0-10)	7 (0-10)	7 (0-10)	0.964
Physical activity (VAS^||^ scale 0-10)	0 (0-10)	0 (0-10)	0.847
Sleep deprivation (VAS^||^ scale 0-10)	0 (0-10)	5 (0-10)	0.987
**Pre-existing health condition**			
Obesity	10 (02)	7 (13)	0.432
Arterial hypertension	13 (25)	9 (16)	0.340
Diabetes mellitus	4 (8)	3 (5)	0.710
Smoking	1 (2)	4 (7)	0.364
Impact of pandemic			0.882^†^
Changes in monthly income	4 (08)	3 (5)	-
Mental health	31 (60)	31 (56)	-
Family concerns	16 (31)	18 (33)	-
GAD^¶^ score	6 (0-18)	6 (5-13)	0.360
GAD^¶^ score ≥5	46 (88)	23 (42)	0.0001
Burnout	45 (86)	34 (62)	0.004
Alcohol consumption	30 (06)	23 (42)	0.123
Tobacco use	5 (10)	4 (7)	0.737
Return to smoking during COVID-19 pandemic	4 (08)	3 (5)	0.710
*Cannabis* use	1 (02)	0 (0)	0.486
Sleeping pills consumption	17 (33)	2 (4)	0.0001
Working with adolescents as patients	36 (69)	37 (67)	0.838

Results are presented in n (%) and median (minimum to maximum values); *PHQ-9 = Patient Health Questionnaire; ^†^Pearson Chi Square; ^‡^RT-PCR = Reverse Transcription Polymerase Chain Reaction; ^§^SARS-CoV2 = Severe Acute Respiratory Syndrome Coronavirus 2; ^||^VAS = Visual Analogue Scale; ^¶^GAD-7 = Generalized Anxiety Disorder

Table 4 demonstrated the univariable and multivariable analysis in the logistic regression model of data reported by 107 pediatric nurses during pandemic related to presence of anxiety. There was only one factor which had significantly increased the chances of anxiety in 4.6 times, adequate quarantine adherence. The univariable and multivariable analysis in the logistic regression model related to presence of depression and burnout showed no statistically significant difference.

**Table 4:** Univariable and multivariable analysis in the logistic regression model of data reported by 107 Pediatric Nurses during pandemic related to presence of anxiety. São Paulo, SP, Brazil, 2020

Independent variables	univariable			multivariable		
	OR^*^	CI^†^ (95%)	p	OR^*^	CI^†^ (95%)	p
Female gender	0,42	0.12-1.47	0.17	3.9	0.55-27.9	0.17
Lack of standardized treatment protocol in pediatric ward	3.2	1.0-10.3	0.05	3.0	0,85-10.7	0.09
Feelings of apprehension	2.4	0.9-6.5	0.09	3.0	0.5-17,9	0.23
Feelings of dissatisfaction	2.5	0.9-7.4	0.10	0.51	0.08-3.3	0.48
Adequate quarantine adherence	1.9	0.8-4.3	0.12	4.6	1.1-20.2	0.04
Arterial hypertension	1.2	0.5-3.3	0.69	-	-	-
PHQ-9^‡^ score ≥10	4.3	1.3-14.0	0.02	2,4	0.5-11.3	0.26
Burnout				1.0	0.19-5.4	0.90
Sleeping pills consumption	3.5	1.0-12.9	0.06	2.3	0.32-16.4	0.41

^*^OR = Odds Ratio; ^†^CI = Confidence Interval; ^‡^PHQ-9 = Patient Health Questionnaire

## Discussion

To our knowledge, this study was relevant in order to investigate physical and mental health issues in Brazilian pediatric nurses during the COVID-19 pandemic in a tertiary hospital. Our study showed relevant impact on the work routine of pediatric nurses, including expressive rates of anxiety, burnout, and depression.

These findings were similar to other studies that evaluated health professionals during COVID-19 pandemic^9^ ^,^ ^18^ ^-^ ^20^, especially female nurses^7^ ^,^ ^21^. In 2004, a study about the SARS outbreak had already revealed increased levels of anxiety and depression. Nowadays, in order to attenuate possible complications, it is necessary to learn from this experience and provide medium or long-term psychological and psychiatric support for these professionals^22^ ^-^ ^24^.

Pediatric nurses reported herein a high rate of burnout, which could have a relationship with increased workload, stress, being single, social distancing and family-associated stress. In fact, the new protocols and routine, impacted their physical and mental health during pandemic COVID-19^25^. In the present study, burnout rate was significantly higher in pediatric nurses, particularly in those who worked with adolescents. This finding may be related to relevant physical and mental impact in adolescent populations during COVID-19 pandemic, particularly in patients hospitalized due to chronic severe disease, requiring multiple immunosuppression therapies^26^. Indeed, a Chinese study showed a higher burnout rate in professionals that worked in usual wards compared to professionals that had worked in the frontline^27^.

We showed high prevalence of burnout in pediatric nurses with anxiety and moderate/severity depression. The reports are linked to emotional exhaustion, anxiety and depression^28^ ^-^ ^30^. Furthermore, stress may be considered as a preceding factor for these mental health issues^29^ ^,^ ^31^. Association between anxiety and depression were also observed in the present study, as also described by other authors^32^ ^-^ ^33^. Depression was significantly mentioned by pediatric nurses with anxiety. These findings were also reported in other studies, possibly related to inadequate available resources, exhaustive workloads, working in tertiary hospitals and in the frontline of COVID-19 pandemic^7^ ^,^ ^25^. 

Furthermore, the lack of other pediatric nurses and other health professionals, as well as lack of standardized treatment protocols, especially in the beginning of pandemic, and quarantine adherence, could also contribute to higher rates of anxiety, burnout and the fear of COVID-19^9^ ^,^ ^25^ ^,^ ^27^. Quarantine adherence involved social distancing, which has been associated with mental health disorders^34^ ^-^ ^35^. Indeed, some studies have found that loneliness was associated with symptoms of depression and anxiety^36^ ^-^ ^37^, and another research has observed that lockdown measures adopted in Germany were associated with higher psychosocial distress and more loneliness, however, they were not related to anxiety and depressive symptoms^38^.

Our study has strengths and limitations. The evaluation of two validated self-reported scales for anxiety and depression, associated to a single item burnout measurement, were extremely relevant to assess the overall mental health impact during this catastrophic period. We also assessed pediatric nurses of a large and referral university hospital. However, limitations included the small sample of pediatric nurses in only one center, lack of information about previous mental health disorders, and the cross-sectional design of the study, allowed us to interpret the results only as current symptoms. The moderate response rate was observed in the present study, possible related to the lack of payment incentive. Furthermore, the absence of a control group with another health professional was another weakness of the present study. In addition, these validated mental health tools were not evaluated in pediatric nurses in other circumstances, such as out of COVID-19 pandemic period.

The COVID-19 pandemic is undoubtedly a stressful experience for different health care providers^39^ ^-^ ^41^. Thus, since the beginning of the COVID-19 pandemic, and in order to care for nurses and other health professionals, our institution has developed a program to offer mental health and psychological/psychiatric support and treatment^42^.

## Conclusion

This study revealed that the mental health of pediatric nurses was undoubtedly impacted during COVID-19 pandemic, corroborating the importance of continuous monitoring of the mental health of professionals. Most of them worked in the frontline of COVID-19, under precarious conditions, working with reduced team and with expressive decrease in their income. Current anxiety was a relevant issue associated with quarantine adherence, which included social distancing by pediatric nurses in the frontline during COVID-19 pandemic. Burnout was also an important mental condition for these professionals that worked with adolescents, reinforcing culture of good teamwork, collaboration practices and psychological/psychiatric approaches. Recognizing mental health status of pediatric nurses is essential to plan future and also for preventive strategies.
